# Perception, concerns, and practice of ChatGPT among Egyptian pharmacists: a cross-sectional study in Egypt

**DOI:** 10.1186/s12913-024-11815-1

**Published:** 2024-11-28

**Authors:** Taha Abd-ElSalam Ashraf Taha, Derar H. Abdel-Qader, Kareem R. Alamiry, Zeyad A. Fadl, Aya Alrawi, Nada K. Abdelsattar

**Affiliations:** 1 Faculty of Medicine, Fayoum University, Fayoum, Egypt; 2 Faculty of Pharmacy and Medical Sciences, The University of Petra, Amman, Jordan; 3 Faculty of Pharmacy, Suez Canal University, Ismailia, Egypt

**Keywords:** ChatGPT, Pharamacists, Perception, Concerns, Practice

## Abstract

**Background:**

The emergence of large language models (LLMs) like ChatGPT attracted significant attention for their potential to revolutionize pharmacy practice. While artificial intelligence (AI) offers promising benefits, its integration also presents unique challenges.

**Objectives:**

This cross-sectional study aimed to explore the current Egyptian pharmacists’ perceptions, practices, and concerns regarding ChatGPT in pharmacy practice.

**Methods:**

The study questionnaire was shared with pharmacists during March and April 2024. We included pharmacists licensed by the Egyptian Ministry of Health and Population. We adapted a convenient sampling technique by sending the research questionnaire via emails, student networks, social media (Facebook and WhatsApp), and student organizations. Any pharmacist interested in participating followed a link to review the study description and was asked to provide electronic consent before continuing with the study. Data were analyzed using SPSS software, employing Chi-square tests for categorical variables and Spearman’s correlation for continuous variables. Statistical significance was set at *p* < 0.05.

**Results:**

The study sample size included 428 pharmacists from the main economic regions of Egypt. The results revealed a strong recognition (73.6%) among participants of ChatGPT’s anticipated benefits within pharmacy practice. Around two-thirds of the participants (65.9%) expressed disagreement or neutrality regarding the application of ChatGPT for analyzing patients’ medical inputs and providing individualized medical advice. Regarding factors affecting perception, we found that the region is the only factor that significantly contributed to the level of perception among pharmacists (*P* = 0.011) with Greater cairo region showing the highest perception level. We found that 73.6% of participants who have heard about ChatGPT reported high levels of concern. One-third of participants never use ChatGPT in their pharmacy work, and 20% rarely use it. Using Spearman’s correlation test, there was no significant correlation between anticipated advantages, concerns and practice level (*P* > 0.05).

**Conclusion:**

This study reveals a generally positive perception of ChatGPT’s potential benefits among Egyptian pharmacists, despite existing concerns regarding accuracy, data privacy, and bias. Notably, no significant associations were found between demographic factors and pharmacists’ perceptions, practices, or concerns. This underscores the need for comprehensive educational initiatives to promote informed and responsible ChatGPT utilization within pharmacy practice. Future research should explore the development and implementation of tailored training programs and guidelines to ensure the safe and effective integration of ChatGPT into pharmacy workflows for optimal patient care.

**Supplementary Information:**

The online version contains supplementary material available at 10.1186/s12913-024-11815-1.

## Introduction

Artificial intelligence (AI) has emerged as a transformative technology with the potential to revolutionize various sectors, including healthcare. The field’s inception can be traced back to the Dartmouth Summer Research Project on AI in 1956, which laid the foundation for subsequent advancements in machine learning and other AI methodologies [1]. Early research focused on developing rule-based systems and expert systems, aiming to mimic human decision-making in specific domains [2]. However, these approaches had limitations in handling complex and nuanced situations.

Artificial intelligence (AI) empowers machines to imitate human intelligence and execute duties that require human intellect [3]. This transformative technology is permeating the healthcare landscape, revolutionizing areas like diagnosis, treatment, and medication management [4, 5]. These advancements have been instrumental in shaping AI’s track and its increasing application across varied domains. Within the Egyptian healthcare system, which faces challenges such as medication shortages, limited access to healthcare in rural areas [6], AI holds significant potential for improving pharmaceutical care and enhancing the role of pharmacists.

Large language models (LLMs) like ChatGPT, a powerful offshoot of AI, are garnering increasing attention for their potential to revolutionize pharmacy practice [7]. The rapid adoption of ChatGPT in educational settings has sparked significant interest in its potential applications across various disciplines, including healthcare. A recent multinational study among university students in Arab countries, including Egyptian students, revealed that nearly half of the participants had heard of ChatGPT, and over half of those had already used it [8].

The convergence of AI and Large language models (LLMs) within pharmacy practice proposes both promising benefits and potential challenges. AI tools are capable of executing routine duties such as medication dispensing and stock control. Consequently, this frees up pharmacists’ time to carry out complex patient care activities, including medication counseling and chronic disease management [9, 10]. Additionally, Large language models (LLMs) like ChatGPT can provide patients with accessible and personalized medication information, answer health-related queries, and support pharmacists in medication adherence counseling [11]. Therefore, pharmacists will be able to provide better patient education, pay more attention to medication safety, and enhance overall care quality.

ChatGPT, exclusively licensed to Microsoft, was tested on the Taiwanese Pharmacist Licensing Examination in 2023 to assess its knowledge in the pharmaceutical domain. Despite achieving higher accuracy in the English version than the Chinese one, ChatGPT ultimately failed the exam, highlighting the limitations of large language models in specialized fields, including potential translation errors and the inability to assess ChatGPT’s performance on image-based questions [12]. Being trained on an enormous database, ChatGPT exhibits remarkable versatility in handling complex language tasks, positioning it as a prime candidate for integration into the pharmacist’s toolkit [13].

Despite these benefits, several concerns arise regarding potential inaccuracies [14] and limitations in understanding complex medical scenarios [15]. In addition, there are ethical issues surrounding data privacy, transparency, and fairness [14, 16]. Striking a careful balance between leverage and caution, with emphasis on thorough training, human oversight, and ethical considerations [14, 17], is crucial to ensure ChatGPT safely complements and empowers pharmacists. Ultimately, this will lead to a future of more efficient, personalized, and inclusive healthcare [18].

Despite the growing global interest in ChatGPT and its potential applications in healthcare, its specific integration within pharmacy practice, particularly in developing countries like Egypt, remains largely unexplored. This presents a significant knowledge gap, as understanding the perceptions, practices, and concerns of pharmacists in these contexts is crucial for responsible and effective implementation of LLMs in healthcare settings. This study addresses this gap by focusing on Egyptian pharmacists’ perceptions, practices, and concerns regarding ChatGPT in their professional settings. This is particularly relevant as Egypt, like many developing countries, faces unique healthcare challenges that could potentially benefit from AI-driven solutions. The findings of this study will not only contribute to the growing body of literature on AI in healthcare but will also provide valuable insights for policymakers, educators, and pharmacy professional organizations in Egypt and similar settings.

## Methods

### Study design

We conducted this cross-sectional study in 2024 over the course of March 1st to April 30th, 2024. We included Egyptian pharmacists which is estimated to be approximately 313,000 pharmacists [19]. The STROBE guidelines were adopted for reporting this study [20].

### Data collection

We recruited collaborators and provided them with a session about the study idea and objectives. Each collaborator was responsible for coltolecting data from 40 pharmacists. Furthermore, we implemented a convenient sampling technique by sending the research questions via emails, student networks, social media (Facebook and WhatsApp), and student organizations. Any pharmacist interested in participating followed a link to review the study description and was asked to approve their participation electronically prior to continuing with the study. We mitigated bias by conducting anonymous surveys, encouraging participants to give honest responses.

### Survey development

The study questionnaire was adapted from the final version of a similar Jordanian study [18]. We contacted the authors for access to the full questionnaire and a linguistic expert has translated the questionnaire into Arabic. We performed a Pilot data collection and analysis during February 2024 to ensure the validity questionnaire’s Arabic version. We asked participants of the pilot study to share their comments and all edits were considered in the Arabic version of the survey by the linguistic expert. To test the reliability of the scales, Cronbach’s alpha reliability test was used to assess the internal consistency of the scales, revealing acceptable values for level of perception (α = 0.65), level of concerns (α = 0.68), and level of practice (α = 0.62).

The survey included four sections (supplementary 1). The first section includes the baseline characteristics of the participants. The second section includes pharmacists’ assessment of the benefits of incorporating ChatGPT into practice. The third section contains comments on the investigation of pharmacists’ concerns about incorporating ChatGPT into clinical practice. In the last section, pharmacists’ practices using ChatGPT are evaluated.

Pharmacists’ perceived benefits, concerns and practice were assessed on a 5-point Likert scale from strongly disagree to strongly agree, scoring 1 to 5, respectively. Scores of perception, concerns and practice levels were categorized into high and low groups according to the 80% bloom limit threshold [21]. Scores for Perception levels ranged from 37 to 85; Scores of 69 to 85 (≥ 80%) were considered high perception levels. Scores for concerns level ranged from 5 to 25, with 21 to 25 (≥ 80%) indicating high concern levels. Scores for practice levels range from 7 to 17, with 15 to 17 (≥ 80%) indicating a high level of practice.

### Sample size calculation

The required sample size was determined with the Raosoft^®^ sample size tool (http://www.raosoft.com/samplesize.html), employing the following formula: n = P×(1 − P)×z^2^ /d^2^. Under a margin of error (d) of 5%, a confidence level of 95% and a response distribution (P) of 50%, the minimum total sample size to be collected is 384 participants.

### Ethical considerations

On 5th of January 2024, this study received ethical approval (approval number R-535) from the research ethics committee at the Faculty of Medicine, Fayoum University, Egypt.

An informed consent was obtained from all the participants. The online questionnaire provided a brief research overview on the first page, followed by a Yes/No question to confirm participation. When a participant replies “YES”, he or she instantly agrees to participate, and the survey begins. All data was anonymized, and participants could not be recognized.

### Statistical analysis

We used IBM SPSS Statistics software (version 29) for data analysis. A combination of inferential and descriptive techniques were used. Frequency and percentage were used for categorical data. Kolmogorov-Smirnov test and histogram visualization were used to test the data for normality. They revealed the non-normality of our data (*p* ≤ 0.05). The mean scores of the perception scale were calculated for each region in Egypt and a heat map was built using Adobe photoshop 2024 software to demonstrate the difference between these regions. This visualization highlights geographic variations in pharmacists’ perceptions of ChatGPT, which can be valuable for identifying areas with greater or lesser acceptance of the technology and informing targeted interventions.

To examine associations between participants’ baseline characteristics and their awareness of ChatGPT, as well as their perception, concerns, and practice levels, the Chi-square test was chosen due to its suitability for analyzing relationships between categorical variables. Given the non-normal distribution of the perception, concerns, and practice scales, Spearman’s rho correlation was employed to assess the correlations between these variables, as it is a non-parametric method appropriate for analyzing relationships between non-normally distributed continuous variables. The significance of results was determined at a *P* value less than 0.05.

## Results

### Sociodemographic characteristics

The study cohort was predominantly composed of young adults, with most participants falling within 20–30 age range, accounting for (85.5%, *n* = 366) of the participants, with a slight male predominance, with (55.6%, *n* = 238) compared to (44.4%, *n* = 190), subsequently. Most participants held a Bachelor’s degree (87.6%, *n* = 375). Regarding the responders’ jobs, the vast majority were community pharmacists (50%, *n* = 214) followed by hospital pharmacies (24.3%, *n* = 104). The degree of AI technology recognition varied among responders. Only a few of them had significant experience applying these technologies within pharmacy practice (2.6%, *n* = 11), while a notable portion reported some relevant experience (22.4%, *n* = 96). The majority indicated some recognition of the technology but away from the pharmaceutical field (55.1%, *n* = 236), and only (19.9, *n* = 85) claimed that they didn’t understand the technology. Self-assessed digital literacy was predominantly high, with most participants rating their technological skills as Good or very Good (77.8%, *n* = 333). Furthermore, most participants indicated that they heard about ChatGPT (81.3%, *n* = 348). Table 1 summarizes participants’ demographics.

**Table 1:** Demographics and general characteristics of the participants (*n* = 428)

Variables	*N*(%)*
Sex	
Female	190 (44.4%)
Male	238 (55.6%)
Age	
20–30	366 (85.5%)
31–40	29 (6.8%)
41–50	25 (5.8%)
More than 51	8 (1.9%)
Region	
The Greater Cairo region	65 (15.2%)
Alexandria region	54 (12.6%)
The Canal region	76 (17.8%)
The Delta region	67 (15.7%)
Central Upper Egypt region	56 (13.1%)
South Upper Egypt region	53 (12.4%)
North Upper Egypt region	57 (13.3%)
Educational level	
Bachelor’s	375 (87.6%)
Master’s	33 (7.7%)
PhD	20 (4.7%)
Profession	
Academic	64 (15%)
hospital pharmacy	104 (24.3%)
Community pharmacy	214 (50%)
others	46 (10.7%)
A prior understanding of technology for artificial intelligence or natural language processing	
None	85 (19.9%)
Some, but not in a pharmaceutical setting	236 (55.1)
Some, in a pharmaceutical setting	96 (22.4%)
Significant experience, in a pharmaceutical setting	11 (2.6%)
proficiency with digital technology and computers (grade yourself)	
Poor	3 (0.7%)
Fair	58 (13.6%)
Good	180 (42.1%)
Very good	153 (35.7%)
Excellent	34 (7.9%)
Have you heard of ChatGPT?	
yes	348 (81.3%)
no	70 (16.4%)
Not sure	10 (2.3%)

^*^Valid percent was used if there were any missing data

### Egyptian pharmacists’ perceptions about ChatGPT

Analysis of perception questions revealed a strong recognition (73.6%, *n* = 315) of ChatGPT’s promising benefits within the pharmacy practice. A substantial majority (73.1%, *n* = 313) acknowledged the potential advantages of ChatGPT utilization for pharmacists and content creation purposes. Similarly, a considerable proportion (65%, *n* = 278) agreed on the utility of ChatGPT for offering academic material for education about pharmaceutical products and different therapies.

Regarding the accuracy of medication information provided by ChatGPT, participant agreement was average (50%, *n* = 214). The application of ChatGPT for analyzing medical history of the patients and giving individualized therapeutic suggestions elicited the most dissent, with a majority of participants (65.9%, *n* = 282) expressing disagreement or neutrality. However, a notable proportion of participants (34.1%, *n* = 146) still expressed agreement with this potential application.

Participant responses also underscored the perceived potential of ChatGPT for equipment performance monitoring and proactive identification of potential issues, with a majority (59.3%, *n* = 254) expressing agreement towards this application. Table 2 summarizes the perceptions toward ChatGPT use.

**Table 2:** Summary of participants’ responses to perception statements (*n* = 428)

Statements	Strongly disagree	Slightly disagree	Neither agree nor disagree	Slightly agree	Strongly agree
pharmacist can benefit from using ChatGPT	1(0.2%)	2(0.5%)	110 (25.7%)	129 (30.1%)	186 (43.5%)
ChatGPT can provide accurate information regarding medicine	47 (11%)	54 (12.6%)	113 (26.4%)	122 (28.5%)	92 (21.5%)
ChatGPT can assist in product training by providing accurate and upto-date information	1 (0.2%)	83 (19.4%)	111 (25.9%)	130 (30.4%)	103 (24.1%)
ChatGPT can be used to provide 24/7 customer support to patients, healthcare providers, and other stakeholders	5 (1.2%)	84 (19.6%)	104 (24.3%)	134 (31.3%)	101 (23%)
ChatGPT can be used to match patients with clinical trials based on their medical history and other criteria	44 (10.3%)	72 (16.8%)	105 (24.5%)	117 (27.2%)	90 (21%)
ChatGPT can be used to provide medical education and training tohealthcare professionals	4 (0.9%)	6 (1.9%)	140 (32.7%)	168 (39.3%)	108 (25.2%)
ChatGPT can be used to analyze patient data and provide personalized treatment recommendations based on their unique medical history and genetic profile	59 (13.8%)	83 (19.4%)	140 (32.7%)	69 (16.1%)	77 (18%)
ChatGPT can assist in content creation by generating ideas for blog posts, social media posts, and other marketing materials	1 (0.2%)	6 (1.4%)	108 (25.2%)	143 (33.4%)	170 (39.7%)
ChatGPT can be used to provide educational content related to your products or therapeutic areas	5 (1.2%)	63 (14.7%)	82 (19.2%)	116 (27.1%)	162 (37.9%)
ChatGPT can assist sales representatives by answering common questions about the products they are promoting	2 (0.5%)	4 (0.9%)	118 (27.6%)	156 (36.4%)	148 (34.6%)
ChatGPT can also be used to create role-playing scenarios where trainees can practice their sales pitch or communication skills in a safe and controlled environment	4 (0.9%)	72 (16.8%)	121 (28.3%)	131 (30.6%)	100 (23.4%)
ChatGPT can be programmed to identify quality control issues during the manufacturing process	4 (0.9%)	10 (2.3%)	127 (29.7%)	153 (35.7%)	134 (31.3%)
ChatGPT can analyze manufacturing data to identify areas where the manufacturing process can be optimized to improve efficiency or quality	3 (0.7%)	72 (16.8%)	107 (25%)	135 (31.5%)	111 (25.9%)
ChatGPT can be used to monitor equipment performance and detect potential issues before they become major problems	16 (3.7%)	59 (13.8%)	99 (23.1%)	149 (34.8%)	105 (24.5%)
ChatGPT can assist in research and development by analyzing data and identifying trends that can lead to the development of new and improved pharmaceutical products	2 (0.5%)	5 (1.2%)	130 (30.4%)	169 (39.5%)	122 (28.5%)
ChatGPT can be used to provide training on regulatory requirements and best practices related to compliance	2 (0.5%)	7 (1.6%)	50 (11.7%)	174 (40.7%)	195 (45.6%)
ChatGPT can monitor and analyze data to identify potential complianceissues, such as deviations from established manufacturing processes or regulatory requirements	5 (1.2%)	14 (3.3%)	116 (27.1%)	163 (38.1%)	130 (30.4%)
ChatGPT can help pharmaceutical companies manage compliance risks by identifying potential compliance issues before they become major problems	1 (0.2%)	9 (2.1%)	128 (29.9%)	158 (36.9%	132 (30.8%)

### Egyptian pharmacists’ concerns about ChatGPT

**Fig. 1:**
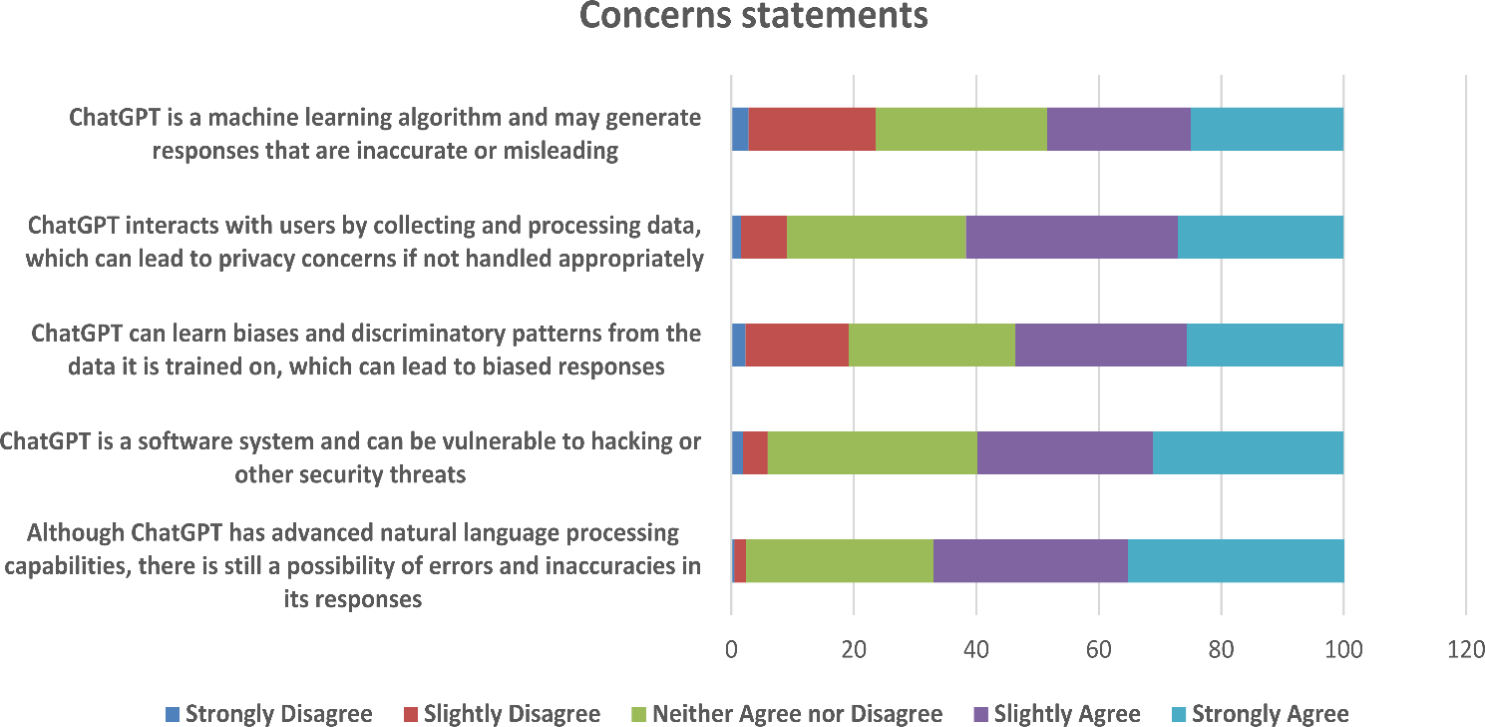
Summary of participants’ responses to concerns statements (*n* = 428)

Significant concerns regarding the potential limitations of ChatGPT were also identified in Fig. 1. A majority of participants (67.1%, *n* = 287) expressed worry about the possibility of errors in the tool’s answers. Confidentiality of data and its protection emerged as significant concerns, with a majority of participants (61.7%, *n* = 264) expressing worries regarding possible privacy breaches, and (59.8%, *n* = 256) highlighting the susceptibility of software systems to security threats or hacking. Concerns regarding how accurately the tool generates answers were also prominent, with a notable proportion (48.4%, *n* = 207) acknowledging the potential for ChatGPT to generate inaccurate or misleading information. Furthermore, about half of the responders (53.5%, *n* = 229) raised concerns about the potential for the tool to perpetuate biases and discriminatory patterns present in training data.

### Practices of pharmacists in using ChatGPT

Table 3 summarizes the use of ChatGPT among our responders in pharmacy practice. Approximately one-fourth of the responders (29.4%, *n* = 126) mentioned no previous use of the tool in pharmacy practice, whereas, (19.9%, *n* = 85) mentioned rare use. As regards recommending the tool to other pharmacists, most of the responders (70.6%, *n* = 302) indicated they would recommend ChatGPT to other pharmacists. Conversely, (17.8%, *n* = 76) remained undecided.

Participant responses regarding the functions they conduct using ChatGPT were diverse. Looking for possible drug–drug interactions was conducted by (30.6%, *n* = 131) of the pharmacists. ChatGPT utilization for acquiring data about drugs and diseases was reported by less than one-third of the participants (30.6%, *n* = 131). Furthermore, ChatGPT utilization for medication reconciliation was limited, with only (7%, *n* = 30) of participants and (25.9%, *n* = 111) for determining appropriate dosage regimens for patients. Adverse drug reaction identification and management using the AI tool were reported by (35.5%, *n* = 152) of participants.

**Table 3:** Summary of participants responses to practice statements (*n* = 428)

Questions	*N*(%)*
Current frequency of using ChatGPT in pharmaceutical areas:	
Never	126 (29.4)
Rarely (once a month or less)	85 (19.9)
Occasionally (2–3 times a month)	128 (29.9)
Frequently (once a week or more)	89 (20.8)
Would you recommend the use of ChatGPT to other pharmacists?	
Yes	302 (70.6)
No	50 (11.7)
Not sure/haven’t decided yet	76 (17.8)
Have you used the ChatGPT to check for Drug-drug interactions?	
Yes	131 (30.6)
No	297 (69.4)
Not sure	0 (0)
Have you ever used the ChatGPT to as drug and disease information source (for example: ask Chat GPT questions about particular conditions, treatments, medications, or lifestyle changes)?	
Yes	131 (30.6)
No	297 (69.4)
Not sure	0 (0)
Have you ever used the ChatGPT for medication reconciliation?	
Yes	30 (7)
No	398 (93)
Not sure	0 (0)
Have you ever used the ChatGPT to determine appropriate dosage regimens for patients?	
Yes	111 (25.9)
No	317 (74.1)
Not sure	0 (0)
Have you ever used the ChatGPT to identify or manage adverse drug reactions?	
Yes	152 (35.5)
No	276 (64.5)
Not sure	0 (0)

### Factors associated with hearing of ChatGPT

As shown in Table 4, sex, age, education, and job were not significantly associated with hearing of ChatGPT (*p* > 0.05). In contrast, the region, previous recognition of AI, and degree of proficient use of technology were significantly associated with the hearing of ChatGPT. The region was significantly associated with hearing of ChatGPT (*p* = 0.05), for example, participants from South Upper Egypt region were more likely to hear of ChatGPT (88.7%, *n* = 47) as compared to those who were from The Delta region (73.1%, *n* = 49). Prior understanding of technology for AI was significantly associated with hearing of ChatGPT (*p* < 0.001), participants who had some Prior understanding of technology for AI in a pharmaceutical setting (87.6%, *n* = 85) were more likely to hear of ChatGPT than those who hadn’t have some previous recognition of AI technology (60%, *n* = 51). Proficiency was significantly associated with hearing of ChatGPT (*p* < 0.001), for instance, participants with very good proficiency (90.8%, *n* = 139) more likely to hear of ChatGPT than participants with poor proficiency (0%, *n* = 0).

**Table 4:** Association between participants’ sociodemographic data and hearing of ChatGPT (*n* = 428)

Variables	Have you heard of ChatGPT?			
	Yes	No	Not sure	P-value
Sex				0.57
Female	152(80)	32(16.8)	6(3.2)	
Male	196(82.4)	38(16)	4(1.7)	
Age				0.482
20–30	300(82)	56(15.3)	10(2.7)	
31–40	24(82.8)	5(17.2)	0(0)	
41–50	19(76)	6(24)	0(0)	
More than 51	5(62.5)	3(37.5)	0(0)	
region				0.05
The Greater Cairo region	57(87.7)	6(9.2)	2(3.1)	
Alexandria region	40(74.1)	13(24.1)	1(1.9)	
The Canal region	66(86.8)	10(13.2)	0(0)	
The Delta region	49(73.1)	15(22.4)	3(4.5)	
Central Upper Egypt region	41(73.2)	11(19.6)	4(7.1)	
South Upper Egypt region	47(88.7)	6(11.3)	0(0)	
North Upper Egypt region	48(84.2)	9(15.8)	0(0)	
Educational level				0.72
Bachelor’s	302(80.5)	63(16.8)	10(2.7)	
Master’s	29(87.9)	4(12.1)	0(0)	
PhD	17(85)	3(15)	0(0)	
Profession				0.2
Academic	57(89.1)	7(10.9)	0(0)	
hospital pharmacy	89(85.6)	15(14.4)	0(0)	
Community pharmacy	166(77.6)	40(18.7)	8(3.7)	
Pharmaceutical manufacturing	9(69.2)	3(23.1)	1(7.7)	
others	27(81.8)	5(15.2)	1(3)	
A prior understanding of technology for artificial intelligence or natural language processing				< 0.001
None	51(60)	32(37.6)	2(2.4)	
Some, but not in a pharmaceutical setting	204(86.8)	26(11.1)	5(2.1)	
Some, in a pharmaceutical setting	85(87.6)	9(9.3)	3(3.1)	
Significant experience, in a pharmaceutical setting	8(72.7)	3(27.3)	0(0)	
proficiency with digital technology and computers (grade yourself)				< 0.001
Poor	0(0)	3(100)	0(0)	
Fair	47(81)	9(15.5)	2(3.4)	
Good	131(72.8)	41(22.8)	8(4.4)	
Very good	139(90.8)	14(9.2)	0(0)	
Excellent	31(91.2)	3(8.8)	0(0)	

All data were presented as N (%). Valid percent was used if there were any missing data. Statistical analysis was done using Chi-square test

### Factors associated with perception, concerns, and practice levels

Table 5 displays the relationship between responders’ sociodemographic characteristics and their perception, concerns, and practice levels. The region was significantly associated with the perception level (*p* = 0.011). For example, participants from Central Upper Egypt region were more likely to have high level of perception (32.1%, *n* = 18) as compared to those from South Upper Egypt region (11.3%, *n* = 6). Moreover, the hearing of the tool was significantly linked to the level of concerns (*p* = 0.05). For example, participants who had not heard of ChatGPT were more susceptable to have high concerns (87.1%, *n* = 61) as compared to those who had heard of it (73.6%, *n* = 256). However, sex, age, educational level, profession, prior understanding of technology for AI, and proficiency with digital technology weren’t linked to participants’ perception, concerns, and practice levels.

**Fig. 2:**
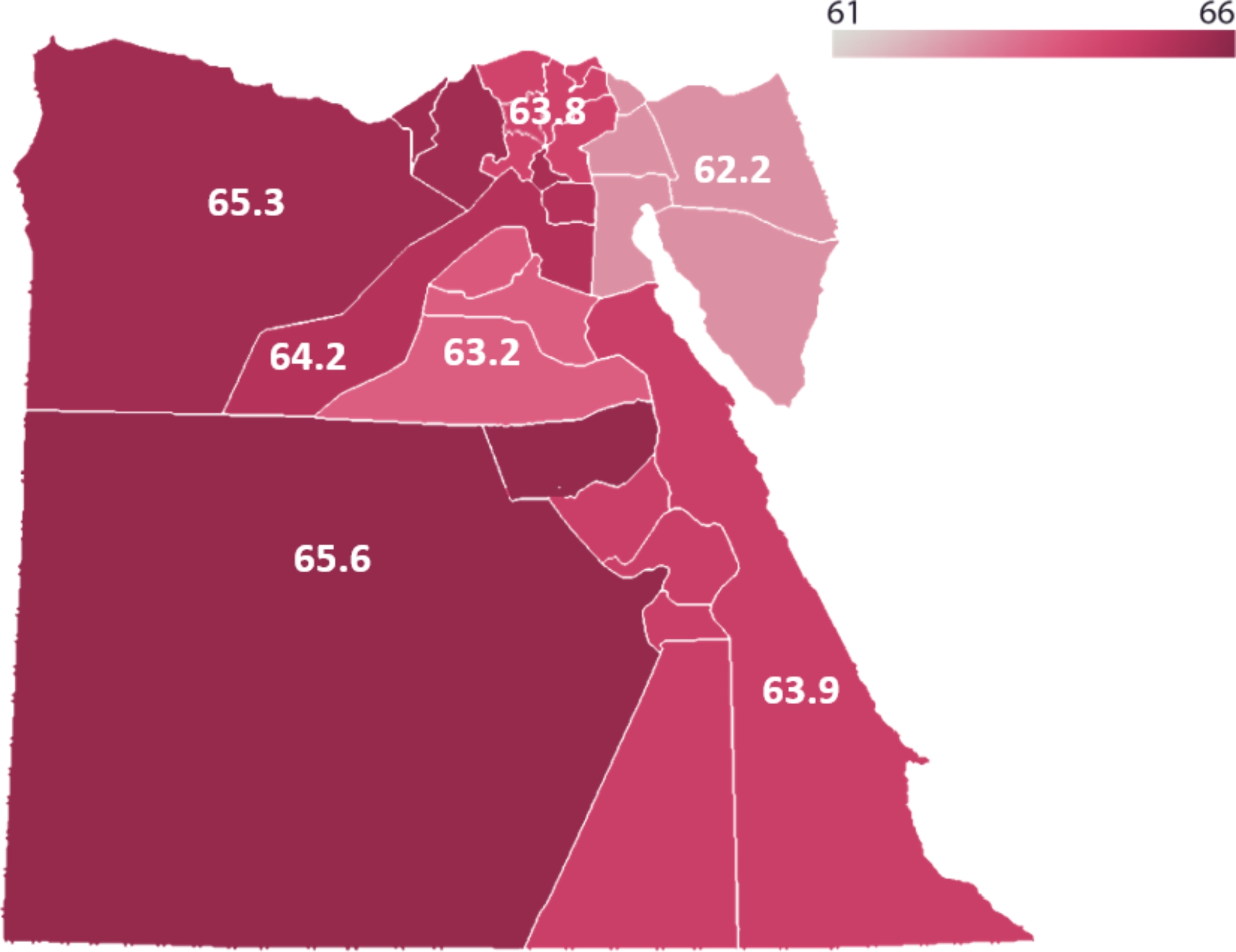
Perception score distribution in main regions of Egypt

The distribution of the mean perception scores across main economic regions of Egypt is shown in Fig. 2. Notably, the Central Upper Egypt region exhibits the highest mean perception score of 65.5, followed by Alexandria region with mean score of 65.3. On the other hand, the Canal region demonstrates the lowest mean perception score of 62.2.

**Table 5:** Association between participants’ sociodemographic data and their perception, concerns, and practice of ChatGPT (*n* = 428)

Variables	Level of perception		*P*-value	Level of Concerns		*P*-value	Level of Practice		Sig.#
	Poor	Good		Low	High		Low	High	
Sex			0.803			0.06			0.9
Female	149 (78.4)	41 (21.6)		54 (28.4)	136 (71.6)		126(66.3)	64(33.7)	
Male	189 (79.4)	49 (20.6)		49 (20.6)	189 (79.4)		159(66.8)	79(33.2)	
Age			0.225			0.77			0.9
20–30	284 (77.6)	82 (22.4)		85 (23.2)	281 (76.8)		242(66.1)	124(33.9)	
31–40	27 (93.1)	2 (6.1)		9(31)	20 (69)		20(69)	9(31)	
41–50	21 (84)	4 (4.4)		7(28)	18(72)		17(68)	8(32)	
More than 51	6 (75)	2 (25)		2(25)	6(75)		6(75)	2(25)	
region			0.011			0.1			0.8
The Greater Cairo region	48 (73.8)	17 (26.2)		19(29.2)	46(70.8)		47(72.3)	18(27.7)	
Alexandria region	38 (70.4)	16 (17.8)		19(35.2)	35(64.8)		38(70.4)	16(29.6)	
The Canal region	67 (88.2)	9 (11.8)		21(27.6)	55(72)		47(61.8)	29(38.2)	
The Delta region	51 (76.1)	16 (23.9)		13(19.4)	54(80.6)		42(62.7)	25(37.3)	
Central Upper Egypt region	38 (67.9)	18 (32.1)		14(25)	42(75)		39(69.6)	17(30.4)	
South Upper Egypt region	47 (88.7)	6 (11.3)		9(17)	44(83)		36(67.9)	17(30.4)	
North Upper Egypt region	49 (86)	8 (14)		8(14)	49(86)		36(63.2)	21(36.8)	
Educational level			0.144			0.675			0.08
Bachelor’s	294 (78.4)	81 (21.6)		88(23.5)	287(76.5)		252(67.2)	123(32.8)	
Master’s	30 (90.9)	3 (9.1)		10(30.3)	23(69.7)		17(51.1)	16(48.5)	
PhD	14 (70)	6 (30)		5(25)	15(75)		16(80)	4(20)	
Profession			0.924			0.178			0.09
Academic	50 (78.1)	14 (21.9)		14(21.9)	50(78.1)		40(62.5)	24(37.5)	
hospital pharmacy	83 (79.8)	21 (20.2)		17(16.3)	87(83.7)		75(72.1)	29(27.9)	
Community pharmacy	167 (78)	47 (22)		58(27.1)	156(72.9)		140(65.4)	74(34.6)	
Pharmaceutical manufacturing	10 (76.9)	3 (23.1)		5(38.5)	8(61.5)		5(38.5)	8(61.5)	
others	28 (84.8)	5 (15.2)		9(27.3)	24(72.7)		25(75.8)	8(24.2)	
A prior understanding of technology for artificial intelligence or natural language processing			0.694			0.183			0.6
None	71 (83.5)	14 (16.5)		27(31.8)	58(68.2)		54(63.5)	31(36.5)	
Some, but not in a pharmaceutical setting	183 (77.5)	53 (22.5)		51(21.7)	184(78.3)		162(68.9)	73(31.1)	
Some, in a pharmaceutical setting	75 (78.1)	21 (21.9)		24(24.7)	73(75)		63(64.9)	34(35.1)	
Significant experience, in a pharmaceutical setting	9 (81.8)	2 (18.2)		1(9.1)	10(90.9)		6(54.5)	5(45.5)	
proficiency with digital technology and computers (grade yourself)			0.817			0.061			0.46
Poor	3 (100)	0(0)		2(66.7)	1(33.3)		1(33.3)	2(66.7)	
Fair	47(81)	11(19)		18(31)	40(69)		38(65.5)	20(34.5)	
Good	142 (78.9)	38(21.1)		35(19.4)	145(80.6)		127(70.6)	53(29.4)	
Very good	121(79.1)	32(20.9)		36(23.5)	117(76.5)		98(64.1)	55(35.9)	
Excellent	25(73.5)	9(26.5)		12(35.3)	22(64.7)		21(61.8)	13(38.2)	
Have you heard of ChatGPT?			0.687			0.05			0.16
yes	274 (78.7)	74 (21.3)		92(26.4)	256(73.6)		239(68.7)	109(31.3)	
no	57 (81.4)	13 (18.6)		9(12.9)	61(87.1)		40(57.1)	30(42.9)	
Not sure	7 (70)	3 (30)		2(20)	8(80)		6(60)	4(40)	

All data were presented as N (%). Valid percent was used if there were any missing data. Statistical analysis was done using Chi-square test

### Investigating the correlation between identified benefits and concerns of ChatGPT implementation

(Supplementary 2) shows that practice level, concerns level, and perception level were not significantly correlated (*p* > 0.05).

## Discussion

This study contributed to Egyptian pharmacists’ perceptions, concerns, and practices regarding the application of ChatGPT in the pharmaceutical field. The majority of participants in this study believed in the benefits of implementing ChatGPT in the field of pharmacy. Our finding that 73.6% of Egyptian pharmacists recognize ChatGPT’s potential benefits aligns with Abu Hammour et al.‘s study in Jordan, where 77.2% of pharmacists held similar views [18]. In our study, the assumption that attracted the highest percentage of agreement is the ability of ChatGPT to train and enhance pharmacists’ practices related to compliance (86%). However, only 57.4% at Abu Hammour et al., agreed with this assumption.

The application of ChatGPT to analyze patients’ data and develop individualized recommendations has been met with the lowest level of agreement among both Egyptian and Jordanian pharmacists (34.1% and 48.2% respectively). These subjective findings contradict other studies. For instance, Qarajeh et al. (2023) has found that ChatGPT may provide individualized therapy solutions in chronic kidney disease patients by processing a wide range of patient data, such as medical histories, blood test results, and concurrent health concerns. These may include precise medicine doses, advised dietary adjustments, changes in daily behaviors, and specialized monitoring techniques [22]. However, Users still lack confidence in ChatGPT due to its limited database of information. Its functionality is dependent on previously learned data and lacks regular update [23]. Therefore, Schork et al., revealed the need to evaluate the utility of AI-based medical products [24]. In addition, Johnson et al., pointed out that the successful translation of AI models into real-world applications depends not only on accuracy, but also on the ability to perform tasks reliably, and safely, and develop personalized health recommendations [25].

Regarding factors affecting perception, we found that the region is the only factor that significantly contributed to the level of perception among pharmacists (*P* = 0.011). Greater Cairo, Alexandria, and Central Upper Egypt were found to be the regions with the highest perception whereas, South Upper Egypt scored the lowest perception scores. This is consistent with the human development index (HDI) which is a scoring system based on measurements of health, education, and income to reflect a country’s overall level of human development and ranks the governments according to the measured scores. Alexandria and Greater Cairo ranked at the top of HDI, while governments of South Upper Egypt had the lowest scores [26].

Pharmacists who work in an academic career have shown the highest percentage of pharmacists who heard about ChatGPT (89.1%). On the other hand, community pharmacists and those working in pharmaceutical manufacturing were the least likely to hear about ChatGPT (77.6% and 69.2% respectively). Usually, postgraduate education is not mandatory for pharmacists, except for those in an academic career. A study conducted among Egyptian pharmacists found that community pharmacists are less likely to hold postgraduate degrees than hospital pharmacies [27]. This may explain why fewer percentages of them have heard about ChatGPT since the tool is most widely used for academic purposes [28].

Prior understanding of AI technologies and proficiency with digital technology significantly contributed to the hearing of ChatGPT (P value < 0.001). This is consistent with the recommendations of UNESCO to implement artificial intelligence within the curriculums for better perception and wise practice [29]. In comparison to other Middle Eastern countries, Egypt hasn’t yet integrated Artificial intelligence systems into curriculums. But recently the Egyptian Council for Artificial Intelligence has proposed the “Egyptian Charter for Responsible AI” which proposes a vision, mission, and goals for applying AI in different sectors such as education, health, etc [30]. In addition, 14.3% of pharmacists in this study have rated their proficiency with digital technology and computers as poor or fair. On a larger scale, 33% of employees in Egypt feel the lack of digital competencies which affects the percentage of people hearing about ChatGPT [31]. Hence, it is imperative to emphasize the integration of essential digital literacy skills into both pre-university and university education, ensuring their incorporation into the mainstream curriculum for all practitioners [32]. The mission of the Egyptian Ministry of Health and population aims to foster coordination between the Ministry and scientific research agencies in the fields of technology and applied health research, aligned with the national plan for scientific and technological research [33].

On the contrary, perceptions and practices were not affected by hearing of ChatGPT, which reflects poor understanding and misconceptions about ChatGPT (*p* > 0.05). Thus, further research should address the sources of participants’ information and try to assess the quality of these sources. Several concerns were detected about ChatGPT use. The probability of errors and inaccuracies in ChatGPT responses due to language processing was the concern that met the highest percentage of agreement among Egyptian pharmacists in this study and Jordanian pharmacists as well (67.1% & 73.3% respectively). Moreover, participants in our study and Abu-Hammour et al., were concerned about privacy issues regarding data collection, performance, and disadvantages of ChatGPT in terms of hacking or other security threats [18]. Possible inaccuracies, mistakes, and moral and confidentiality issues are also reported in the context; To prevent plagiarism, editing by human authors is essential [34]. “Nature” magazine pointed out that artificial intelligence can facilitate the creation of spam, ransomware, and other malicious products [35]. ChatGPT can also provide false information by extracting messages from invalid sources [36]. Artificial intelligence may not always give the right answer, especially in complex medical queries, and is only as good as the information it learns. If the training data is biased, the model will also be biased [37]. Likewise, a study has reviewed the use of ChatGPT in drug discovery, but it remained doubtful about the utilization of AI in medication and noted the need for more discussion, straightforwardness, and user instructions [38]. These concerns emphasize the need for cautious use, security measures, and consistent checking of artificial intelligence frameworks such as ChatGPT.

In our study, we found that 73.6% of participants who have heard about ChatGPT reported high levels of concern. In agreement with our results, Abu Hammour et al., found that most respondents who have heard of ChatGPT will be more concerned than those who are unsure or have never heard of it. This relationship can be credited to the spread of deception around AI tools. Given the rapid development of artificial intelligence and its increasing integration into various fields, misunderstandings, and misstatements are not uncommon. For example, people who have known ChatGPT but have not yet used it may get wrong or misleading messages, having concerns about its quality, pressure, performance, or potential risk. This wrong information can be from many sources, including questionable online blogs, hearsay, or misunderstandings about the capabilities and limitations of technology. Hence, giving exact, clear, and exact data about ChatGPT and its possible uses in culture is vital to address these concerns and correct mistaken assumptions.

Concerning the use of ChatGPT in clinical practice, approximately half of Abu-Hamour 2023 participants reported that they have never used ChatGPT in clinical practice, and approximately 29% said they used it rarely (e.g., once a month). Our numbers are even better; one-third never use ChatGPT in their pharmacy practice, and 20% rarely use it. Additionally, while about 73.6% of our participants recommended the use of ChatGPT to other pharmacists, only half of the participants at the Jordanian study recommended using ChatGPT [18]. A study conducted in China found that physicians employed ChatGPT for various tasks including medication review (*P* = 0.0089), patient medication instructions (*P* = 0.0032), adverse drug reactions (ADR) analysis (*P* = 0.0483), ADR causality assessment (*P* = 0.023) and drug counseling (*P* = 0.0791). Moreover, they found that most ChatGPT users who have looked for drug-drug interactions and drug interactions based on drug and disease information found ChatGPT to be as effective as or equal to and as effective or more effective than other conventional sources (UpToDate, Medscape, medical journals, etc.) and human decision making [39].

We didn’t observe a significant correlation between perception, concerns and practice levels. This might raise the incidence of ChatGPT misuse. For instance, one may think that ChatGPT replaces his role as a pharmacist not just assist for better performance and this may negatively influence patients’ health. On the contrary, the Jordanian study has reported a significant positive correlation between the perception and concerns levels (*p* < 0.001).

### Strength and limitations

This study represents the first comprehensive evaluation of Egyptian pharmacists’ perceptions, practices, and concerns about ChatGPT. The study gives valuable insights that will help pharmacists and policymakers in Egypt and other countries around the world to better understand the pattern of ChatGPT use and understanding in pharmaceutical practice and thus allow them to identify the boundaries and solutions to its possible comprehensive execution in the future. Moreover, it provides a foundational understanding of Egyptian pharmacists’ perceptions, concerns, and practices regarding ChatGPT, with implications for the future of AI integration in pharmacy practice. The generally positive perception of ChatGPT’s potential benefits, coupled with concerns about accuracy, data privacy, and bias, underscores the need for comprehensive educational initiatives targeting Egyptian pharmacists, focusing on enhancing digital literacy, promoting critical evaluation of AI-generated information, and addressing ethical considerations. These initiatives should include tailored training programs providing practical guidance on utilizing ChatGPT safely and effectively for tasks such as drug information retrieval, patient education, and workflow optimization. Furthermore, regulatory bodies should collaborate with pharmacy professionals and AI experts to establish clear guidelines and regulations for AI use in pharmacy practice, addressing data privacy, security, accuracy, transparency, and accountability. Integrating AI concepts and skills into pharmacy curricula is essential to prepare future pharmacists for the increasing role of AI in their profession, including providing education on the fundamentals of AI, its applications in pharmacy, ethical considerations, and the development of critical evaluation skills.

The results of this study should be considered with caution regarding some limitations. The data was collected online; hence, the questionnaire was only completed by pharmacists who were able to participate from the Internet or had an account on social media. Additionally, a convenient sample was targeted that may not represent all Egyptian pharmacists. While we employed appropriate statistical methods for analyzing our data, it is important to acknowledge certain limitations. The use of a cross-sectional design precludes establishing causal relationships between variables. We can only identify associations, and further research is needed to explore potential causal links. Additionally, the reliance on self-reported data through online surveys may be subject to recall bias and social desirability bias, potentially affecting the accuracy of responses. Finally, the categorization of perception, concerns, and practice levels based on the 80th percentile threshold, although a common practice, introduces a degree of arbitrariness. Different thresholds might yield slightly different results. Future research could explore alternative methods for categorizing these variables or utilize continuous scales for a more nuanced analysis.

### Recommendations

To be clear, Prior to widespread adoption of ChatGPT in pharmaceutical healthcare, a thorough examination of its limitations, ethical and legal implications, data accuracy, and potential for bias is essential. It is recommended to organize awareness campaigns about how to wisely use and benefit from ChatGPT. It is worth noting that awareness campaigns should be targeted to all age categories, both males and females with different academic backgrounds because these factors haven’t shown differences in the level of perception, practice, or concerns. A study among Egyptian nurses and medical managers has found significant differences between their perception levels after they conducted awareness sessions [40]. Consequently, awareness campaigns should be tailored according to the different specialties within the medical field. To facilitate responsible ChatGPT implementation within Egyptian pharmacy practice, we further recommend developing specialized training programs for pharmacists. These programs should focus on enhancing digital literacy, addressing ethical considerations related to AI use in healthcare, and providing practical guidance on utilizing ChatGPT safely and effectively for tasks such as drug information retrieval, patient education, and workflow optimization. Additionally, regulatory bodies should collaborate with pharmacy professionals and AI experts to establish clear guidelines and regulations for AI use in pharmacy. In the future, it’s recommended to use alternative sampling methods, such as stratified random sampling, to achieve a more representative sample of Egyptian pharmacists. Moreover, we recommend the implementation longitudinal studies to assess changes in perceptions and practices over time.

## Conclusion

In conclusion, this study provides valuable insights into Egyptian pharmacists’ perceptions, concerns, and practices regarding ChatGPT. Our findings reveal a generally positive outlook on the potential of AI tools to facilitate pharmacy practice and enhance pharmacists’ knowledge. While pharmacists expressed some concerns about accuracy, data privacy, and bias, the anticipated benefits highlight a willingness to adopt ChatGPT, pending appropriate guidance and safeguards. This underscores the need for comprehensive educational initiatives and clear regulatory guidelines to promote responsible ChatGPT utilization within Egyptian pharmacy practice. By addressing these needs, the potential of ChatGPT to enhance medication safety, improve patient care, and optimize pharmacy workflows can be realized while mitigating potential risks. Thus, ChatGPT should be used with caution, ensuring the information it provides is accurate. Awareness campaigns should be conducted for a better understanding of how to make the most cautious use of the tool. Future research should be conducted to explore barriers and limitations to the use of AI tools in healthcare settings.

## Electronic supplementary material

Below is the link to the electronic supplementary material.

Supplementary Material 1.

## Data Availability

The datasets used and/or analysed during the current study are available from the corresponding author on reasonable request.

## Abbreviations

LLMs: Large language models
AI: Artificial intelligence
HDI: Human development index
ADR: Adverse drug reactions
