# Supplementary material for: Perception, concerns, and practice of ChatGPT among Egyptian pharmacists: a cross-sectional study in Egypt
Source: BMC Health Serv Res. 2024 Nov 28;24:1500. doi: 10.1186/s12913-024-11815-1 (PMC11605968; doi:10.1186/s12913-024-11815-1)
Supplement: Supplementary file 1 — Supplementary Material 1. [file 12913_2024_11815_MOESM1_ESM.docx]

| Spearman’s rho |  |  | Practice level | Concerns Level | Perception level |
| --- | --- | --- | --- | --- | --- |
|  | **Practice level** | **Correlation coefficient** | **1** | **0.04** | **-0.001** |
|  |  | **p-value** |  | **0.4** | **0.98** |
|  | **Concerns score** | **Correlation coefficient** | **0.04** | **1** | **0.03** |
|  |  | **p-value** | **0.4** |  | **0.5** |
|  | **Perception score** | **Correlation coefficient** | **-0.001** | **0.03** | **1** |
|  |  | **p-value** | **0.98** | **0.5** |  |

Table 1 Correlation between perception, concerns, and practice levels
